# Clinical Outcomes for Gastric Cancer following Adjuvant Chemoradiation Utilizing Intensity Modulated versus Three-Dimensional Conformal Radiotherapy

**DOI:** 10.1371/journal.pone.0082642

**Published:** 2014-01-09

**Authors:** Gene-Fu F. Liu, Ryan J. Bair, Eric Bair, Stanley L. Liauw, Matthew Koshy

**Affiliations:** 1 Department of Radiation and Cellular Oncology, University of Chicago Medical Center, Chicago, Illinois, United States of America; 2 Departments of Endodontics and Biostatistics, University of North Carolina-Chapel Hill, Chapel Hill, North Carolina, United States of America; 3 Department of Radiation Oncology, University of Illinois Hospital, Chicago, Illinois, United States of America; University of Algarve, Portugal

## Abstract

**Purpose/Objective(s):**

To determine if intensity modulated radiation therapy (IMRT) in the post-operative setting for gastric cancer was associated with reduced toxicity compared to 3D conformal radiation therapy (3DCRT).

**Materials/Methods:**

This retrospective study includes 24 patients with stage IB-IIIB gastric cancer consecutively treated from 2001–2010. All underwent surgery followed by adjuvant chemoradiation. Concurrent chemotherapy consisted of 5-FU/leucovorin (n = 21), epirubicin/cisplatin/5FU (n = 1), or none (n = 2). IMRT was utilized in 12 patients and 3DCRT in 12 patients. For both groups, the target volume included the tumor bed, anastomosis, gastric stump, and regional lymphatics.

**Results:**

Median follow-up for the entire cohort was 19 months (range 0.4–8.5 years), and 49 months (0.5–8.5 years) in surviving patients. The 3DCRT group received a median dose of 45 Gy, and the IMRT group received a median dose of 50.4 Gy (p = 0.0004). For the entire cohort, 3-year overall survival (OS) was 40% and 3-year disease free survival (DFS) was 41%. OS and DFS did not differ significantly between the groups. Acute toxicity was similar. Between 3DCRT and IMRT groups, during radiotherapy, median weight lost (3.2 vs. 3.3 kg, respectively; p = 0.47) and median percent weight loss were similar (5.0% vs. 4.3%, respectively; p = 0.43). Acute grade 2 toxicity was experienced by 8 patients receiving 3DCRT and 11 receiving IMRT (p = 0.32); acute grade 3 toxicity occurred in 1 patient receiving 3DCRT and none receiving IMRT (p = 1.0). No patients in either cohort experienced late grade 3 toxicity, including renal or gastrointestinal toxicity. At last follow up, the median increase in creatinine was 0.1 mg/dL in the IMRT group and 0.1 mg/dL in the 3DCRT group (p = 0.78).

**Conclusion:**

This study demonstrates that adjuvant chemoradiation for gastric cancer with IMRT to 50.4 Gy was well-tolerated and compared similarly in toxicity with 3DCRT to 45 Gy.

## Introduction

Gastric cancer (GC) accounts for 738,000 deaths worldwide and 10% of total annual cancer deaths [1]. Approximately 20,000 Americans will be diagnosed with GC this year, half of whom are expected to die from the disease [2]. Thus, the development of effective treatments with limited toxicity remains an area of active interest.

In 2001, the Intergroup 0116 randomized trial demonstrated both a relapse-free and overall survival (OS) benefit for the addition of postoperative chemoradiotherapy over surgery alone [3]. However, the benefit of radiotherapy (RT) is tempered by its acute and late effects on adjacent vital organs, highlighting the importance of developing techniques able to spare normal tissues [4].

One such technique is Intensity Modulated Radiation Therapy (IMRT), which utilizes intensity-modulated “beamlets” to conform dose away from vital organs and more closely towards tumor. In comparison, three-dimensional conformal radiotherapy (3DCRT) uses beams of uniform intensity, offering less freedom in sculpting dose around the tumor. Prior dosimetric studies in GC suggest the superior conformality of IMRT may reduce liver and kidney radiation doses [5], [6]. However, whether these dosimetric advantages translate into meaningful clinical improvement is an area of ongoing research.

The purpose of this study is to identify patients with GC who underwent adjuvant chemoradiotherapy and report the comparative outcomes of those treated with IMRT versus 3DCRT.

## Materials and Methods

Our study was approved by the institutional review boards of the University of Chicago Medical Center, University of Illinois at Chicago Medical Center, and the Unversity of Illinois at Chicago Cancer Center. All data was anonymized and individual patient consent was not required as a waiver for the need for written informed consent was approved by the institutional review board of each participating institution.

### Patients

From October 2001 to January 2011, 24 consecutive patients with Stage IB-IIIC GC or gastroesophageal junction cancer were treated with adjuvant RT at the University of Chicago (UCMC) and University of Illinois at Chicago Medical Center (UIMC).

### Treatment

All patients underwent surgical resection. Surgery consisted of subtotal gastrectomy in 12 patients, total gastrectomy in 10 patients, and esophagogastrectomy in 2 patients. The majority of patients received 1 cycle of 5-fluorouracil (5FU) and leucovorin followed by concurrent 5FU/leucovorin with radiation.

Radiation was delivered utilizing IMRT in 12 patients and 3DCRT in 12 patients. For both groups, the planning target volume (PTV) included the tumor bed, anastomosis, gastric stump, and regional lymphatics. In patients who received 3DCRT, the total dose prescribed was 45 Gy in 1.8 Gy daily fractions. In patients who underwent IMRT, four patients received a dose of 45 Gy, seven patients received 50.4 Gy and one patient received 54 Gy due to the presence of a positive surgical margin. Radiation plans were developed on Eclipse (Varian, Palo Alto, U.S.A.) for 14 patients, Pinnacle (Philips, Amsterdam, Netherlands) for 5 patients, and CORVUS (Nomos, Pittsburgh, U.S.A.) for 5 patients. 3DCRT plans were delivered using 3 or more fields. Patients were staged according to American Joint Committee on Cancer (AJCC) 7th Edition 2010 tumor-node-metastasis (TNM) Classification [7].

### Data Collection

Maximal acute and late toxicity grade was scored according to RTOG Acute Morbidity Scoring Criteria and Toxicity Criteria and RTOG/EORTC Late Radiation Morbidity Scoring Schema, respectively, with late toxicity defined as any toxicity occurring greater than 3 months after treatment completion [8]. Acute toxicities were recorded primarily from on-treatment visit notes, while late toxicities were collected across departmental follow-up notes.

Patients were seen at least once weekly while receiving RT, at which point they were evaluated for acute toxicities. Patients were then seen 4–6 weeks following treatment completion. Afterwards, they were followed every 3–6 months for the first 2 years, and every 6–12 months for the next 3 years. Imaging and bloodwork were drawn at similar intervals.

### Statistics

Clinical-pathologic variables of the 2 cohorts were statistically analyzed using JMP version 9 (SAS Institute). All tests of statistical significance were two-sided, and significance was defined as a value of *p*<0.05. The log rank test was used to compare toxicities between IMRT and 3DCRT. Survival estimates were obtained using the Kaplan-Meier method. Outcome parameters were defined as: OS: time of surgery to time of death or last known follow-up; disease free survival (DFS). All events were calculated using standard life table methods, and the differences were compared using Cox regression models.

To estimate the power to detect an association using a Cox proportional hazards model, two simulated data sets of size 12 were generated. The first simulated data set was exponentially distributed with rate parameter 1, and the second simulated data set was exponentially distributed with rate parameter log(HR), where HR is the target hazard ratio. Censoring times were also generated as exponential random variables such that approximately 50% of the data points were censored. This process was repeated 10,000 times for each target hazard ratio. For each of the 10,000 simulated data sets, a Cox proportional hazards model was used to test the null hypothesis of no difference between the two groups. The power was estimated to be the number of times the p-value associated with this null hypothesis was less than 0.05.

## Results

Median follow up for the entire cohort was 19 months (range 0.4–8.5 years), and 49 months (0.5–8.5 years) in surviving patients. Median follow up was similar in those receiving IMRT vs. 3DCRT (24.3 vs. 16.0 months, p = 0.63).

Table 1 describes patient and tumor characteristics. There were no statistically significant differences in mean age, gender distribution, tumor grade, and TNM or overall AJCC stage.

**Table 1:** Patient Characteristics.

	3DCRT (n = 12)	IMRT (n = 12)	*p*-value
Mean age (years)	64	56	0.23
Gender			0.64
Male	10	8	
Female	2	4	
Tumor grade*			0.59
Grade 1	1	2	
Grade 2	5	2	
Grade 3	6	6	
T stage			0.90
1	1	1	
2	7	5	
3	3	5	
4	1	1	
N stage			0.23
0	2	0	
1	1	5	
2	4	3	
3	4	4	
4	1	0	
AJCC stage			0.53
IB	3	1	
IIA	0	1	
IIB	2	4	
IIIA	5	4	
IIIB	1	2	
IIIC	1	0	

AJCC = American Joint Committee on Cancer; 3DCRT = 3-dimensional conformal radiotherapy; IMRT = intensity modulated radiotherapy.

Two patients in the IMRT group did not have tumor grade documented in pathology report.

Table 2 describes treatment characteristics. The 3DCRT group received a median dose of 45 Gy, and the IMRT group received a median dose of 50.4 Gy (p = 0.0004). Otherwise, both groups shared a statistically similar distribution of type of surgery received, surgical margin status, extent of node dissection, type of concurrent chemotherapy.

**Table 2:** Treatment Characteristics.

		3DCRT (n = 12)	IMRT (n = 12)	*p*-value
Surgery Type	Esophagogastrectomy	2	0	0.58
	Total gastrectomy	5	5	
	Subtotal gastrectomy	5	7	
Margin Status	Negative	6	7	0.30
	<3 mm	3	0	
	Positive	3	4	
	Not recorded	0	1	
Extent of node dissection	D1	3	3	0.63
	D2	8	4	
	Not recorded	1	5	
Concurrent chemotherapy	None	1	1	1.00
	5FU/leucovorin	10	11	
	ECF	1	0	
Radiotherapy dose				0.0004¥
	45 Gy	12	4	
	≥50.4 Gy	0	8	

3DCRT = 3-dimensional conformal radiotherapy; IMRT = intensity modulated radiotherapy; ECF = epirubicin, cisplatin, 5FU.

Difference between median dose.

**Figure 1:**
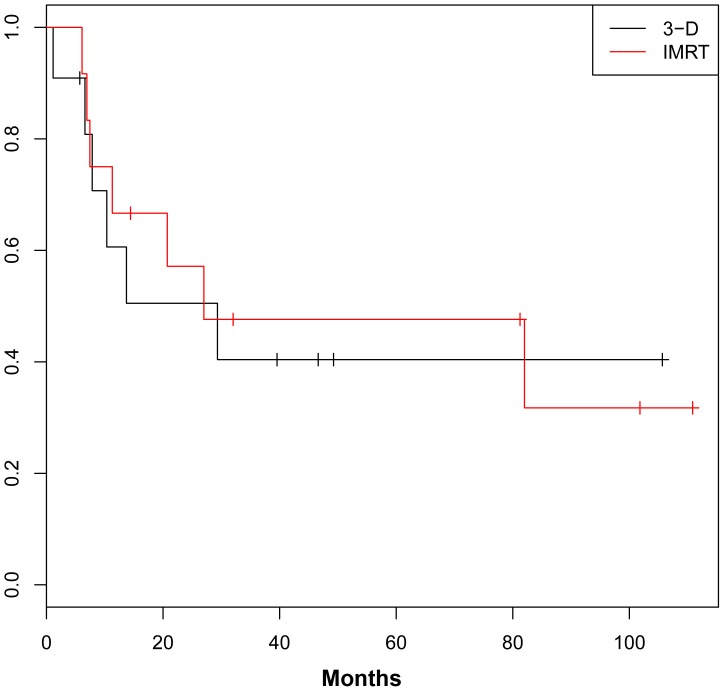
Disease-Free Survival.

**Figure 2:**
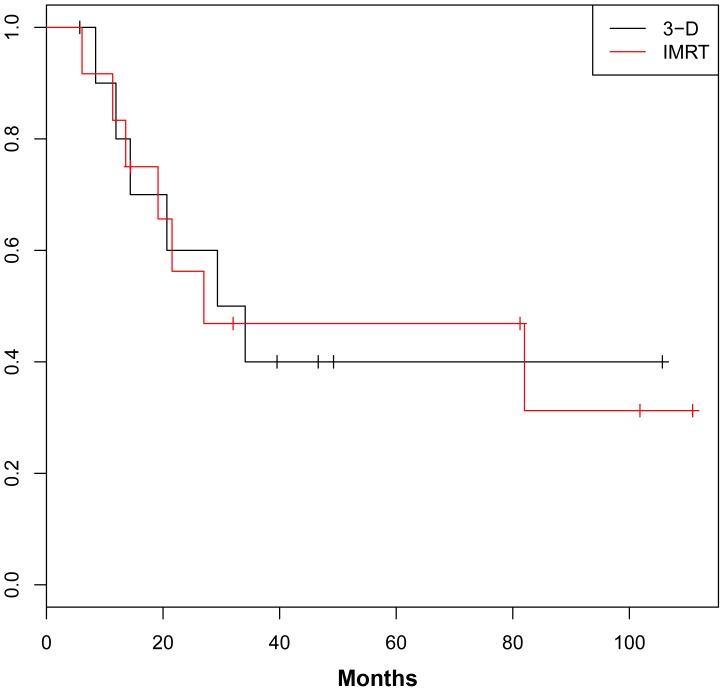
Overall Survival.

**Figure 3:**
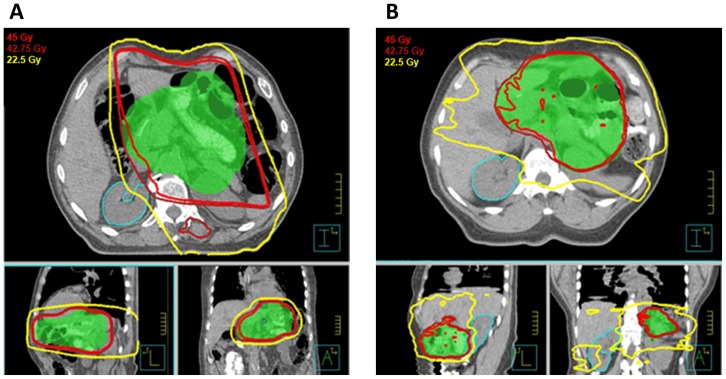
A. 79 year-old gentleman with gastric adenocarcinoma, T1N1M0, who underwent subtotal gastrectomy with D2 nodal dissection and adjuvant chemoradiotherapy with 3DCRT to a dose of 45 Gy. Figure 3B. 64 year-old woman with gastric adenocarcinoma, T2bN2M0, who underwent gastrectomy with D2 nodal dissection. Ten of 14 nodes were positive and she had positive proximal and distal surgical margins. She then received adjuvant chemoradiotherapy with IMRT to a dose of 45 Gy, with a 5.4 Gy boost to the anastomosis.

For the entire cohort, 3-year DFS was 40.6% (Figure 1) and 3-year OS was 40.0% (Figure 2). OS and DFS were similar between the IMRT and 3DCRT groups.

Table 3 describes RT-related acute and late toxicity. Regarding acute toxicities, median weight lost from the first to last week of RT was 3.2 kg in the 3DCRT group and 3.3 kg in the IMRT group (p = 0.47). The median percent weight loss over the same period was also similar (5.0% in the 3DCRT group and 4.3% in the IMRT group, p = 0.43). There were two acute grade 3 toxicities. One patient who received 3DCRT became feeding-tube dependent, and one patient who received IMRT required esophageal dilatation 1 month after completing RT. Acute normal tissue toxicities are detailed in Table 4.

**Table 3:** Acute Radiation Morbidity.

	3DCRT (n = 12)	IMRT (n = 12)	*p*-value
Median weight loss through radiotherapy (kg)	3.2	3.3	0.47
Median percent body weight loss through radiotherapy	5.0%	4.3%	0.43
No. patients with acute grade 2 toxicities	8	11	0.32
No. patients with acute grade 3 toxicities	1	0	1.00
No. patients with late grade 2 toxicities	1	3	0.59
No. patients with late grade 3 toxicities	0	0	NS
Median serum creatinine increase (mg/dL)	0.1	0.1	0.78

**Table 4:** RTOG Acute Radiation Morbidity Grade Detail.

		Grade 2	Grade 3	Total grade ≥2
		(No. patients)	(No. patients)	(No. patients)
Pharynx and esophagus	IMRT	3	1	4
	3DCRT	4	1	5
Upper gastrointestinal	IMRT	9	0	9
	3DCRT	4	0	4

Regarding long term toxicities, there were no patients in either cohort, who experienced grade 3 long term renal toxicity. At last follow up, the median increase in serum Cr was 0.1 mg/dL in the IMRT group and 0.1 mg/dL in the 3DCRT group (p = 0.78). There were no late grade 3 gastrointestinal toxicities in either group. Late normal tissue toxicities are detailed in Table 5.

**Table 5:** EORTC/RTOG Late Effects Grade Detail.

		Grade 2	Grade 3	Total grade ≥2
		(No. patients)	(No. patients)	(No. patients)
Esophagus	IMRT	1	0	1
	3DCRT	0	0	0
Small/Large Intestine	IMRT	0	0	0
	3DCRT	1	0	1
Kidney	IMRT	3	0	3
	3DCRT	1	0	1
Liver	IMRT	0	0	0
	3DCRT	0	0	0

## Discussion

This study compares the outcomes of patients with GC treated with postoperative IMRT versus a similar cohort treated with postoperative 3DCRT. Overall, toxicity rates were comparable between cohorts, though patients receiving IMRT received a higher median dose. There were no differences between cohorts in regards to OS or DFS.

Prior series have suggested a potential role for IMRT in the adjuvant treatment of GC. In 2009, investigators from Germany reported the outcomes of two sequentially treated GC cohorts, with 27 patients treated with 3DCRT from 2001–2005, and 33 patients treated with IMRT from 2002–2007; the majority of both the 3DCRT and IMRT groups received 45 Gy (68% vs. 91%, respectively, p = NS) [9]. Median OS (18 months vs. not reached, p = 0.0492) and DFS (13 months vs. not reached, p = 0.0216) favored the IMRT cohort. Actuarial 2-year OS also statistically favored the IMRT group (67% vs. 37%, p = 0.0492). Dosimetric parameters suggested that this benefit may be attributed to IMRT allowing for larger PTVs (mean 1,397 vs. 1,768 cm^3^, p = 0.0368), while maintaining lower V30 parameters for the left (mean 26.8% vs. 19.5%, p = 0.0015) and right kidneys (mean 11.6 vs. 15.6%, p = 0.0170). Reduced renal irradiation was associated with statistically lower creatinine 6 weeks post-RT for the IMRT group (mean 0.71 vs. 0.84 µmol/L, p = 0.0210). However, creatinine values were similar at last follow-up and no late renal toxicity grade 3–4 (LENT-SOMA scale) was observed in either cohort. Of note, the results of this study are complicated by the two groups having received varied chemotherapy regimens, with 96% of the 3DCRT group receiving 5FU/folinic acid and 70% of the IMRT group receiving oxaliplatin/capecitabine (p<0.0001).

A more recent series from Stanford University also demonstrated IMRT to have a more favorable toxicity profile [10]. In 26 patients who received 3DCRT versus 31 patients who received IMRT, more patients receiving 3DCRT required a treatment break (3 days vs. 0, no p-value reported). Regarding late toxicity, at a median follow-up of 1.4 years, the median serum creatinine was unchanged for patients treated with IMRT (0.80 mg/dL), whereas it had increased 0.20 mg/dL in those receiving 3DCRT (0.80 to 1.0 mg/dL, p = 0.02). However, IMRT was not associated with a clear dosimetric advantage, as the median kidney V20 was statistically similar between the IMRT and 3D CRT groups (17.5% vs. 22%, respectively, p = 0.17). As in our series, differences in hepatotoxicity between those receiving 3DCRT versus IMRT were not detected, though median liver V30 was reduced in the IMRT group (16.1 and 28%, p<0.001). In contrast to the previously discussed series from Germany, disease outcomes between the 3DCRT and IMRT groups were similar; 2-year OS (51 vs. 65%, p = 0.5), DFS (60% vs. 54%, p = 0.8), and local control (83% vs. 81%, p = 0.9), respectively.

In this study, there were no detected differences in toxicity or disease control despite the IMRT cohort having received a higher median dose (50.4 Gy vs. 45 Gy, p = 0.0004) than the 3DCRT cohort. This dose is also higher than that received by the IMRT cohorts of the two previously discussed studies. Potentially, if our entire 3DCRT cohort was prescribed 50.4 Gy, then indeed toxicity rates may have been different. This is suggested by our prior dosimetric analysis, in which patients were planned to receive 50.4 Gy using either two- or three-field 3DCRT versus IMRT [6]. Compared with the three-field plan, IMRT significantly reduced liver V30 dose (63.6% vs. 18.9%, p = 0.010), as well as right kidney V20 dose (20.9% vs. 11.6%, p = 0.027).

Dosimetric studies analyzing the feasibility of IMRT vs. 3DCRT in GC suggest that neither modality is categorically superior, but that individual patient anatomy should dictate the choice. In a study from the University of Toronto where both 5-field 3DCRT and IMRT plans were created and evaluated by gastrointestinal radiation oncologists, IMRT was preferred in 17 of 19 cases (89%) [11]. Similarly while IMRT was dosimetrically rated higher for kidney sparing in 69% of the reviewers' ratings, conversely 31% considered 3DCRT better able to spare the kidneys. Therefore, it stands to reason that the decision between the two modalities should likely be individualized. Figures 3A and 3B pictorially highlight the dosimetric characteristics of 3DCRT and IMRT, respectively, in two patients treated adjuvantly for GC. While 3DCRT generally spreads low-dose radiation across a smaller body volume, IMRT better conforms high-dose radiation towards tumor and away from normal tissues.

When neither modality is dosimetrically superior, 3DCRT should be encouraged, as it holds other advantages over IMRT. 3DCRT typically irradiates a smaller volume of normal tissue by distributing its dose through fewer beams, has less interleaf scatter dose, and utilizes fewer monitored units. In addition, 3DCRT may also better treat tumors subject to intrafraction motion. The sub-diaphragmatic location of the gastric bed subjects it to respiratory motion, and investigators from Fudan University recently reported a mean superior-inferior intrafraction respiratory motion of 11.1 mm in 22 patients treated with postoperative RT for GC [12]. Because IMRT delivers dose through smaller beam apertures, there is an increased risk of intrafraction miss for patients whose tumors move significantly. Finally, there are substantial medical cost savings when using 3DCRT over IMRT. These differences should encourage the individualized evaluation of both modalities.

Our study is limited by biases inherent in any retrospective review. However, in contrast to prior series, our study population was not preferentially treated with IMRT despite its availability. Rather, patients were treated with IMRT and 3DCRT contemporaneously, with 8 of 12 patients treated with 3DCRT after 2006, potentially eliminating bias associated with treating across different eras. Another study limitation is that a range of tumor stages/grades were included in our patient population, though there was no statistical difference between groups.

Furthermore, we performed a retrospective power analysis, determining our study size has a power of 0.12 to detect a hazard ratio of at least 1.5 in regards to overall survival. Thus, our study is not powered to reveal significant differences between the groups regarding disease outcomes. However, we believe our results remain worthwhile, as they further quantify the clinical magnitude of nephrotoxicity post-RT, agreeing with prior series that median creatinine increases are small, an important consideration for a patient population that may require systemic therapy in the future. Additionally, our results reiterate that should IMRT reduce nephrotoxicity, this advantage may also be small; in the series from Stanford, creatinine increased by 0.0 mg/dL for IMRT vs. 0.2 mg/dL for 3DCRT (p = 0.02), and in the series from Germany, there was no statistical difference in creatinine at 1 year or at last follow-up between RT modalities. In our series, both groups experienced a median creatinine increase of 0.1 mg/dL (p = 0.78). Furthermore, while kidneys do not respond acutely to radiation, we believe our median follow-up of 19 months should capture radiation nephropathy, as such changes have been detected at 6 months [4]. Finally, we believe our results are noteworthy, as they agree with available dosimetric studies. As noted in the series from Stanford reporting reduced creatinine levels with IMRT, V20 and mean kidney radiation doses were not statistically lowered with IMRT. Instead, IMRT was associated with a significant increase in right mean kidney dose (11.9 Gy vs 10.54 Gy, p = 0.04). Finally, given the decline of GC incidence and that less than half of patients eligible are currently referred for adjuvant RT [13], future retrospective series on this topic will likely feature small numbers of patients as well.

In conclusion, this analysis revealed that patients treated with IMRT to a total dose of 50.4 Gy tolerated their treatment well and had long term outcomes similar to a cohort treated with 3DCRT to 45 Gy. The decision between IMRT and 3DCRT will vary by patient anatomy, for which individual comparison plans should be considered.
